# Real-world evidence for clergy well-being: developing a culturally grounded self-screening tool to promote sustainable mental health outcomes

**DOI:** 10.3389/fpsyg.2026.1677071

**Published:** 2026-03-03

**Authors:** Wen-Cheng Li, Yu-Ting Chin, Li-Hsin Wu, I-Ling Ling, Chien-Hung Lee

**Affiliations:** 1 Department of Public Health, College of Health Sciences, Kaohsiung Medical University, Kaohsiung, Taiwan; 2 Department of Psychology, University of Toronto, Toronto, ON, Canada; 3 Department of Marketing & Tourism Management, National Chiayi University, Chiayi, Taiwan; 4 Research Center for Precision Environmental Medicine, Kaohsiung Medical University, Kaohsiung, Taiwan; 5 Department of Medical Research, Kaohsiung Medical University Hospital, Kaohsiung Medical University, Kaohsiung, Taiwan

**Keywords:** Chinese clergy, clergy well-being, psychological well-being, scale development, spiritual well-being, subjective well-being, work-related psychological health

## Abstract

**Introduction:**

Clergy well-being (CWB), encompassing subjective, psychological, and spiritual dimensions, is critical for sustaining work-related psychological health (WPH). However, dominant well-being models are largely grounded in Western individualistic assumptions and lack cultural sensitivity to collectivist contexts such as Chinese-speaking communities. Despite facing distinct vocational and spiritual stressors, clergy remain underrepresented in occupational health psychology research. This study aimed to develop and validate a culturally grounded self-screening tool for CWB and to examine a dual-spectrum model of WPH among Chinese clergy.

**Methods:**

A mixed-method design was conducted in Taiwan across two studies. Study 1 involved item development, expert content validation, and exploratory factor analysis with 150 clergy. Study 2 included confirmatory factor analysis, measurement invariance testing, and structural equation modeling with 437 clergy to validate the factor structure and examine the predictive relationships between CWB dimensions (subjective, psychological, spiritual well-being) and WPH outcomes (engagement, stability, fatigue, burnout).

**Results:**

The final 34-item scale demonstrated strong internal consistency and construct validity. Analyses supported a three-factor structure of CWB and a four-factor structure of WPH. Subjective well-being showed the strongest and most consistent associations with engagement, stability, fatigue, and burnout. Psychological well-being significantly reduced fatigue and enhanced stability, whereas spiritual well-being primarily predicted engagement and stability. Gender measurement invariance was established.

**Discussion:**

This study provides a culturally sensitive and psychometrically validated self-screening instrument for assessing clergy well-being and work-related psychological health in Chinese contexts. By integrating collectivist cultural values and spiritual dimensions into occupational health psychology, the findings advance culturally inclusive well-being theory and inform targeted interventions. The accompanying online self-assessment tool enhances practical applicability and supports ongoing well-being monitoring.

## 1 Introduction

Clergy well-being (CWB) is a multidimensional construct encompassing the physical, psychological, and spiritual health of those serving in pastoral roles (Proeschold-Bell et al., 2013). Clergy often navigate blurred boundaries between their personal and professional lives while facing intense demands for moral leadership and spiritual guidance. Although their vocation can provide deep fulfillment, it also exposes them to significant psychological stressors, including burnout, anxiety, and depression (Proeschold-Bell et al., 2013; Milstein et al., 2020; Essa-Hadad et al., 2022). Biblical teachings that emphasize self-sacrifice (e.g., Luke 14:26–27) may inadvertently intensify these burdens (Lee and Fung, 2023). Further, congregational expectations and the “fishbowl” effect of constant public scrutiny compound these challenges (Rowatt, 2001).

In occupational health psychology, well-being is central to promoting health, safety, and fulfillment in the workplace (Warr, 1990). However, the term is often used without conceptual precision. Sinclair et al. (2024) address this ambiguity by proposing a 2 × 2 typology that distinguishes between hedonic well-being (pleasure-focused) and eudaimonic well-being (meaning-focused), across both general and work-specific domains. This framework offers an integrated understanding of how general life satisfaction (Quadrants 1 and 2) interacts with work-related outcomes such as engagement and burnout (Quadrants 3 and 4).

Yet, this typology remains grounded in Western values of individualism and self-expression (Markus and Kitayama, 1991). From the perspective of Geert Hofstede’s national culture framework, Chinese societies are generally characterized by lower individualism, higher power distance, stronger long-term orientation, and greater restraint, which shape how well-being is experienced and expressed (Hofstede, 2001; Hofstede et al., 2010). In such contexts, collectivism, hierarchical relationships, and persistent mental health stigma may limit the applicability of Western assumptions (Ng, 1997; Hui et al., 2014). Cultural pressures discourage the open acknowledgment of psychological distress, and clergy may underreport symptoms due to their perceived spiritual, moral, and communal responsibilities (Li, 2005). These dynamics necessitate culturally sensitive frameworks that reflect the lived realities of Chinese clergy. In response, this study extends existing typologies by incorporating spiritual well-being (SpWB) alongside subjective well-being (SWB) and psychological well-being (PWB), conceptualizing well-being as a set of inner resources that foster resilience, growth, and vocational flourishing.

In parallel with these conceptual developments, real-world evidence has emerged as an essential tool in public health, particularly for informing sustainable mental health policy and system design. Real-world evidence, which derives from naturalistic and community-based settings, is especially valuable for populations like Chinese clergy who are both underserved by formal health systems and difficult to reach through conventional research approaches. By developing a culturally sensitive self-screening tool, the present study contributes to a real-world evidence base that may guide both individual-level interventions and broader policy decisions aimed at promoting clergy well-being within restrictive and culturally complex environments.

## 2 Literature review

### 2.1 Dimensions of clergy well-being

The World Health Organization defines well-being as a multidimensional construct, typically encompassing physical, mental, and social domains (World Health Organization, 1948). However, not all dimensions are equally relevant across roles or cultures. This study focuses on subjective well-being (SWB; Diener, 1984), psychological well-being (PWB; Ryff and Sarason, 1989), and spiritual well-being (SpWB; Ellison, 1983), as these best reflect the internal experiences central to clergy life. These domains capture life satisfaction, emotional resilience, and spiritual fulfillment—core elements of well-being in religious vocations. Narrowing the focus enhances the model’s clarity, cultural sensitivity, and practical value for clergy-specific assessment.

This focus is especially appropriate for Chinese clergy, whose well-being is shaped by the demands of spiritual leadership and the influence of collectivist cultural values. Clergy face unique challenges such as emotional labor, isolation, and high spiritual expectations, often in settings where mental health issues are stigmatized. SWB, PWB, and SpWB offer a meaningful lens through which to assess well-being in ways that resonate with clergy roles and reduce barriers to self-reflection and support. SWB refers to individuals’ evaluations of life satisfaction, positive affect, and low negative affect (Diener, 1984). Diener et al. (1999) reviewed three decades of research on SWB indicated that factors such as personality traits, adaptation processes, goal pursuit, and coping strategies significantly influence individuals’ happiness and life satisfaction. Robbins and Hancock (2015) found that SWB provides insight into clergy’s ability to manage emotional and pastoral demands. However, cultural constructs of the self-influence how SWB is expressed. In collectivist cultures like China, relational well-being and harmony with others are central (Lu, 2008).

PWB empathizes with optimal mental health rooted in eudaimonic theories that emphasize personal growth and living authentically (Ryff and Sarason, 1989; Ryff and Singer, 1996). High PWB is linked to greater resilience and lower anxiety and depression (Ryff and Keyes, 1995; Keyes et al., 2010). The model also integrates hedonic (pleasure-focused) and eudaimonic (purpose-focused) approaches to mental health (Cooke et al., 2016). For clergy, PWB is essential in managing emotional and spiritual demands. Strong purpose and environmental mastery foster resilience and stress management (Pargament, 2001). Yet, cultural expectations of perfection and stigma around mental health limit help-seeking among Chinese clergy (Wong et al., 2017; Lou, 2016). These factors make PWB crucial for sustaining clergy well-being and vocational resilience.

SpWB is an important component of clergy well-being, integrating both personal faith and vocational identity. Defined by Ellison (1983) as “the affirmation of life in a relationship with God, self, community, and environment that nurtures and celebrates wholeness,” For clergy specifically, Proeschold-Bell et al. (2014) identified two dimensions to capture both personal and professional expressions of spirituality: ‘Presence and Power of God in Daily Life’ and ‘in Ministry’. SpWB supports emotional resilience and protects against burnout, depression, and occupational distress. Research highlights its benefits: clergy high in SpWB report greater engagement and lower burnout (Parker and Martin, 2011), reduced stress (Rogers, 2023), and enhanced well-being linked to early religious socialization (Upenieks, 2023). Cultural factors like role ambiguity, overload, and stigma surrounding emotional struggle affect how SpWB influences clergy health in Chinese settings (Lee and Fung, 2024; Milstein et al., 2020). These dynamics emphasize the need for culturally tailored assessments that reflect Chinese values such as community, hierarchy, and collectivism.

### 2.2 Dual-spectrum model of work-related psychological health (WPH)

Work-related psychological health (WPH) encompasses both positive and negative workplace perceptions. Warr (1987)and Warr (1990) model organizes emotional states along two axes: anxiety–contentment and depression–enthusiasm, capturing the dual nature of well-being at work. Positive workplace experiences, such as engagement, job satisfaction, and perceived job stability, play a protective role by buffering stress and promoting overall well-being (Daniels et al., 1997; Ford et al., 2011). For instance, Ford et al. (2011) found that job satisfaction enhances performance and reduces burnout, while Leijten et al. (2015) observed that engagement supports mental health. Stable employment and a sense of job security also contribute to workforce retention and psychological resilience (Hobfoll, 1989). In line with these findings, Schaufeli et al. (2002) demonstrated that engagement and burnout are opposite but related experiences with burnout involving tiredness and negativity, and engagement reflecting energy and enthusiasm.

In contrast, negative work experiences such as fatigue and burnout present negative perceptions. Fatigue can result from long working hours, role ambiguity, and spiritual burdens (Maslach and Leiter, 2008; Ricci et al., 2007). Burnout, in particular, is driven by chronic emotional demands, blurred work–life boundaries, and long-standing relational ties to congregations (Maslach and Leiter, 2008; Hobfoll, 1989; Greenhaus and Beutell, 1985). Irregular work hours and role overload further contribute to fatigue and reduced well-being (Ricci et al., 2007). While burnout has been widely documented among clergy (Maslach and Leiter, 2008), fatigue remains underexplored—despite its recognized impact on health and productivity (Ricci et al., 2007). These dynamics highlights the need for an integrated model of WPH that acknowledges both flourishing and strain.

For clergy, this duality is especially important. Although a strong sense of calling can energize ministry work, it may also contribute to fatigue and burnout (Clinton et al., 2017). Francis and colleagues have extensively studied clergy WPH. Their development of the Scale of Emotional Exhaustion in Ministry (SEEM) and the Francis Burnout Inventory (FBI) provided tools to assess both burnout and job satisfaction. Their findings consistently show that job satisfaction mitigates emotional strain and supports psychological resilience (Francis et al., 2004, 2011, 2019). Studies also highlight the role of personality (Francis et al., 2009), gender-specific stress (Robbins et al., 2012), and social support (Shaw et al., 2021) in shaping clergy WPH. Job satisfaction emerges as a consistent buffer against burnout, making it essential for sustaining long-term vocational health.

This study aligns with vocational health psychology, which views WPH not just as the absence of distress but as a sign of vocational flourishing—marked by energy, meaning, and sustainable well-being. Drawing on Warr’s framework, Sinclair et al. (2024) highlight the need to balance work demands with internal resources. Clergy lacking emotional support are especially prone to burnout. Thus, this study adopts a dual-spectrum model to explore work- WPH and examine how SWB, PWB, and SpWB help or hinder clergy in managing strain and sustaining engagement.

### 2.3 Significance of the study

This research aims to: (1) develop a culturally relevant clergy well-being (CWB) scale that incorporates subjective well-being, psychological well-being, and spiritual well-being; (2) construct a dual-spectrum model of work-related psychological health (WPH) that reflects positive and negative dimensions; (3) integrate a validated self-screening tool to help Chinese-speaking clergy to self-assess their CWB and WPH; and (4) explore the predictive effects of CWB on WPH outcomes.

## 3 Methods

### 3.1 Procedure and participants

**Figure 1:**
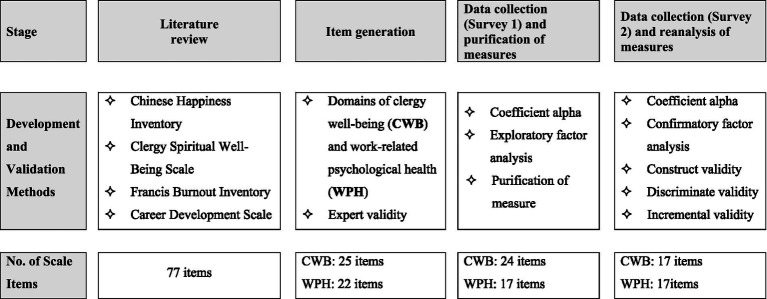
Flow chart of the scale development procedure.

This study contributes to workplace well-being by addressing gaps in understanding CWB in collectivist societies. Scale development followed established models (DeVellis and Thorpe, 2022; Churchill, 1979; Carlson et al., 2000) and used both qualitative and quantitative methods. Initial items were derived from literature reviews and expert interviews. Two surveys were conducted to validate the scale using a systematic development process (see Figure 1).

### 3.2 Study 1: purification of measures and hypotheses testing

#### 3.2.1 Item generation and content validation

Initial CWB items were collected from the Chinese Happiness Inventory (Lu, 1998) and Clergy Spiritual Well-Being Scale (Proeschold-Bell et al., 2014); WPH items were drawn from the Francis Burnout Inventory (Francis et al., 2004), Career Development Scale (Lin, 1989) and Copenhagen Burnout Inventory (Kristensen et al., 2005). Eight experts in scale development, Christian studies, and clergy in the workplace reviewed item clarity, relevance, and coverage. Using Lawshe’s (1975) method, only items with a Content Validity Ratio (CVR) ≥ 0.75 were retained, reducing the pool from 77 to 47 items (see Table 1).

**Table 1:** Original items of clergy well-being (CWB) and Work-related psychological health (WPH) scales.

Scale (items)	CVR
Clergy well-being (CWB: 25 items)	
CWB 01. I frequently feel connected to God’s presence and strength	1.00
CWB 02. My life is filled with wonderful people and things	0.75
CWB 03. I often feel that God’s will and guidance are present in my life	1.00
CWB 04. I find joy and satisfaction in my life	1.00
CWB 05. I discover fulfillment in my life.	1.00
CWB 06. I find a sense of achievement in my life	1.00
CWB 07. I often feel an excellent relationship with God	1.00
CWB 08. I have many joyful memories from my past	0.75
CWB 09. I believe my efforts will be rewarded.	1.00
CWB 10. I actively engage in all aspects of my life	1.00
CWB 11. When leading the worship, I feel the power of God’s presence	1.00
CWB 12. In gatherings, I often experience God’s presence	0.75
CWB 13. I feel mentally refreshed and full of vitality	1.00
CWB 14. I look forward to the future with optimism	0.75
CWB 15. I frequently experience joy and excitement	1.00
CWB 16. In times of crisis among friends, I often feel God’s support	0.75
CWB 17. I have a clear understanding of life’s significance	1.00
CWB 18. I frequently smile and exude positivity	0.75
CWB 19. I perceive the love and presence of God when administering the sacraments.	1.00
CWB 20. I value who I am	1.00
CWB 21. I perceive myself as a confident and appealing individual	1.00
CWB 22. Throughout my life, I regularly experience God’s guidance and power	1.00
CWB 23. I experience a strong sense of security mentally	1.00
CWB 24. I experience a deep sense of happiness	1.00
CWB 25. I can manage my time well and accomplish what I want to do	1.00
Work-related psychological health (WPH 22 items)	
WPH 01. I have good relationships with members, keeping my work stable	1.00
WPH 02. I value my service work	0.75
WPH 03. I am honored by my ministry	1.00
WPH 04. I take great pride in my service work	1.00
WPH 05. When facing low achievement, I reduce my commitment	1.00
WPH 06. Regarding my service work, I just want peace of mind and do not expect too much	1.00
WPH 07. I feel that I cannot bear the heavy pressure of service work	1.00
WPH 08. It is difficult for me to make a breakthrough in my current service	1.00
WPH 09. I firmly remain unwaveringly optimistic about my ministry	1.00
WPH 10. I feel competent with my current ministry.	1.00
WPH 11. Fellow members acknowledge the quality of my service work	1.00
WPH 12. Fellow members always respect me	1.00
WPH 13. Facing the current service-related issues makes me feel lost.	1.00
WPH 14. Repetitive tasks in my job make it uninteresting	1.00
WPH 15. I am unable to derive a sense of accomplishment from the service.	1.00
WPH 16. My workload is heavy and overwhelming.	0.75
WPH 17. There are many conflicts among organizational members and my co-workers.	1.00
WPH 18. Pressure feels overwhelming and stifling	0.75
WPH 19. I feel powerless and unable to live up to my expectations.	1.00
WPH 20. I am passionate about the work of the preach	1.00
WPH 21. I am honored in my ministry job.	1.00
WPH 22. I am not satisfied with the current working situation.	1.00

CVR, content validity ratio.

#### 3.2.2 Data collection (Survey 1) and purification of measures

One hundred and fifty clergy were recruited via convenience and snowball sampling from diverse clergy networks across Taiwan, ensuring demographic and denominational diversity. Exploratory Factor Analysis (EFA) was conducted following the guidelines of Hair et al. (2019), recommending a subject-to-variable ratio of at least 5:1. A sufficient sample size was ensured to meet the minimum requirements for reliable factor extraction.

#### 3.2.3 Item reduction and exploratory factor analysis

Data were analyzed using SmartPLS 4.0 (CB-SEM) with a 4-point Likert scale (1 = strongly disagree, 4 = strongly agree). Sampling adequacy was confirmed via the Kaiser-Meyer-Olkin (KMO) measure. Factors were retained based on eigenvalues > 1, loadings ≥ 0.50, and cross-loading differences ≥ 0.15 (DeVellis and Thorpe, 2022).

### 3.3 Study 2: data collection and reanalysis of measures (Survey 2)

#### 3.3.1 Data collection

A total of 2,466 email addresses were obtained from a national church directory website,^1^ and invitation letters containing a survey link were sent to clergy. The letter indicated that respondents would receive a book from the first author as a token of appreciation. This outreach yielded a sample of 437 clergy (response rate: 17.72%), exceeding the minimum required for confirmatory factor analysis (CFA). Using PLS-SEM–specific criteria, the minimum required sample size for detecting medium-sized effects ranges from approximately 126 to 196 observations (Kock and Hadaya, 2018). As the achieved sample size exceeded this range, the data are sufficient for PLS-SEM analysis.

#### 3.3.2 Construct validity and discriminant validity

CFA was conducted to validate the factor structure identified through EFA. CFA was performed to examine the hypothesized seven-factor structure of the CWB and WPH scales. Model fit was evaluated using established criteria, including the comparative fit index (CFI), Tucker–Lewis index (TLI), and root mean square error of approximation (RMSEA), following recommendations by Hair et al. (2019). Items were retained based on standardized factor loadings and multicollinearity diagnostics. Specifically, items with outer loadings below 0.70 or variance inflation factors (VIF) exceeding 3 were excluded from the final model. Composite Reliability (CR) values above 0.70 were considered indicative of satisfactory internal consistency. Average variance extracted (AVE) values greater than 0.50 were considered indicative of acceptable convergent validity. Discriminant validity was evaluated using the Fornell–Larcker criterion, which compares the square root of AVE to the inter-construct correlations (Fornell and Larcker, 1981).

#### 3.3.3 Measurement invariance

To assess consistency across groups, measurement invariance testing was conducted on the 437 clergy, divided by gender. Using bootstrap multigroup CFA and the Welch-Satterthwaite test, we evaluated the equivalence of the seven-factor model across genders.

#### 3.3.4 Criterion-related and incremental validities explained by WPH and SWB measures

Concurrent and predictive validity were assessed using Pearson’s correlation coefficients and hierarchical regression analyses. To test incremental validity, we examined whether PWB and SpWB added predictive power beyond SWB in relation to work-related psychological health (WPH) outcomes: engagement, stability, fatigue, and burnout.

#### 3.3.5 Common variance bias

To assess common method bias (CMB), a bifactor model was employed as recommended by Reise et al. (2007). In this approach, each observed item was specified to load on both its respective substantive factor and a general method factor capturing shared variance. The model was estimated separately for the CWB and WPH constructs. Model comparisons were conducted between the bifactor model and the original baseline model using chi-square difference testing (Δχ^2^). A nonsignificant Δχ^2^ was used as the criterion to indicate that CMV was not a serious concern.

#### 3.3.6 The effects of CMB on WPH

Structural equation modeling (SEM) was employed to assess the relationships between the three dimensions of CWB and the four domains of WPH. Prior to conducting SEM, univariate normality of all observed variables was assessed using skewness and kurtosis indices.

### 3.4 Ethics statement

It was conducted in accordance with the Declaration of Helsinki and approved by the IRB of Kaohsiung Medical University Hospital [IRB No. KMUHIRB-E(I)-20220144, approved August 10, 2022]. To ensure participant anonymity, written informed consent was waived.

## 4 Results

### 4.1 Study 1: purification of measures

#### 4.1.1 Distribution of Survey 1 and purification of measures

The sample included 57.3% male, 76.7% married, and 68.7% from metropolitan areas; 54.7% had under 10 years of ministry experience (see Table 2). To refine the item pool, EFA was conducted on data, achieving acceptable ratios: 6:1 for 25 CWB items, and 6.8:1 for 22 WPH items (Hair et al., 2019).

**Table 2:** Demographic characteristics in Surveys 1 and 2.

Variables	Survey 1 (*N* = 150)	Survey 2 (*N* = 437)
	Development study	Validation study
	*n* (%)	*n* (%)
Gender		
Male	86 (57.3)	197 (45.1)
Female	64 (42.6)	240 (54.9)
Marital status		
Married	115 (76.7)	338 (77.3)
Single	22 (14.7)	71 (16.2)
Remarried	6 (4.0)	9 (2.1)
Widowed/Divorced	3 (2.0)	19 (4.3)
Location		
Metropolitan city	103 (68.7)	299 (68.4)
Sub-metropolitan city	9 (6.0)	27 (6.2)
Township	19 (12.7)	69 (15.8)
Rural area	10 (6.7)	29 (6.6)
Aboriginal area	9 (6.0)	10 (2.3)
Work experience		
Under 10 years	82 (54.7)	168 (28.5)
11–20 years	29 (19.3)	113 (25.9)
21–30 years	15 (10.0)	74 (16.9)
Over 30 years	24 (16.0)	81 (18.5)

NA, not available.

#### 4.1.2 Item reduction and exploratory factor analysis

EFA identified three factors for CWB and four factors for WPH, with cumulative variances of 67.1 and 65.2%, respectively. All subscales showed strong internal consistency (*α* > 0.7). The factors were labeled: (1) SWB, (2) PWB, and (3) SpWB; the WPH factors were: (1) engagement, (2) stability, (3) fatigue, and (4) burnout (see Table 3). These findings confirmed the model structure.

**Table 3:** Exploratory factor analysis for CWB and WPH scales in Survey 1 (*N* = 150).

Clergy well-being (CWB: 24 items)	Mean	FL	Eigenvalue	Variance	CV
Factor 1: SWB (8 items)			3.512	14.6%	14.6%
CWB 02	3.207	0.654			
CWB 04	3.260	0.680			
CWB 05	3.447	0.659			
CWB 06	3.300	0.702			
CWB 09	3.073	0.693			
CWB 10	3.260	0.660			
CWB 17	3.333	0.584			
CWB 25	2.980	0.607			
Factor 2: PWB (8 items)			7.273	30.3%	44.9%
CWB 13	3.107	0.713			
CWB 14	3.233	0.687			
CWB 15	3.073	0.725			
CWB 18	3.053	0.648			
CWB 20	3.273	0.736			
CWB 21	3.027	0.584			
CWB 23	3.287	0.656			
CWB 24	3.200	0.810			
Factor 3: SpWB (8 items)			5.329	22.2%	67.1%
CWB 01	3.370	0.683			
CWB 03	3.373	0.683			
CWB 07	3.447	0.621			
CWB 11	3.387	0.664			
CWB 12	3.440	0.750			
CWB 16	3.307	0.722			
CWB 19	3.380	0.612			
CWB 22	3.367	0.752			

Work-related psychological health (WPH 17 items)	Mean	FL	Eigenvalue	Variance	CV
Factor 1: Engagement (4 items)			2.947	17.3%	17.3%
WPH 04	3.460	0.633			
WPH 09	3.200	0.730			
WPH 20	3.227	0.801			
WPH 21	3.420	0.791			
Factor 2: Stability (4 items)			2.363	13.9%	31.2%
WPH 01	3.380	0.680			
WPH 02	3.580	0.548			
WPH 11	3.213	0.798			
WPH 12	3.227	0.785			
Factor 3: Fatigue (5 items)			3.574	21.0%	52.3%
WPH 05	1.847	0.634			
WPH 13	2.033	0.652			
WPH 14	1.773	0.807			
WPH 15	1.760	0.787			
WPH 22	1.933	0.768			
Factor 4: Burnout (4 items)			2.193	12.9%	65.2%
WPH 16	0.799	0.799			
WPH 17	0.722	0.722			
WPH 18	0.651	0.651			
WPH 19	0.583	0.583			

FL, factor loading; CV, cumulative variance.

### 4.2 Study 2: data collection and reanalysis of measures (Survey 2)

#### 4.2.1 Distribution of Survey 2

The demographic profile closely resembled that of Survey 1: 54.9% female, 77.3% married, and 68.4% residing in metropolitan areas. Work experience was evenly distributed, with 28.5% reporting less than 10 years of service (see Table 2).

#### 4.2.2 Construct validity and discriminant validity

The model fitting of CFA met established criteria (CFI = 0.989, TLI = 0.985, RMSEA = 0.041; Hair et al., 2019). CFA supported the seven-factor structure for both the CWB and WPH scales. Five items were removed due to low outer loadings (< 0.70) and high multicollinearity (VIF > 3), resulting in 17 items per scale. The final model demonstrated strong fit indices (CFI = 0.989, TLI = 0.985, RMSEA = 0.041). CR was high, ranging from 0.873 to 0.901 for the CWB scale and 0.861 to 0.893 for the WPH scale, confirming internal consistency. Convergent validity was also supported, with AVE values ranging from 0.541 to 0.652 (see Table 4). As shown in Table 5, the AVE for each construct (diagonal values) exceeded the corresponding inter-construct correlations (off-diagonal values), indicating satisfactory discriminant validity. This validation approach is consistent with prior work in public health instrument development, such as the SISQ, which emphasized strong internal consistency and factorial validity across related psychological constructs (Li et al., 2020).

**Table 4:** Assessment of construct validity of CWB and WPH scales in Survey 2 (*N* = 437).

Clergy well-being (CWB: 17 items)	Mean	OL^a^	AVE	α	CR
Factor 1: SWB (4 items)			0.594	0.856	0.856
CWB 04	3.307	0.787			
CWB 05	3.563	0.822			
CWB 06	3.229	0.738			
CWB 17	3.494	0.754			
Factor 2: PWB (5 items)			0.628	0.891	0.893
CWB 13	3.027	0.834			
CWB 14	3.190	0.825			
CWB 20	3.265	0.802			
CWB 21	3.050	0.654			
CWB 23	3.229	0.826			
Factor 3: SpWB (8 items)			0.570	0.914	0.916
CWB 01	3.396	0.755			
CWB 03	3.471	0.769			
CWB 07	3.355	0.833			
CWB 11	3.362	0.695			
CWB 12	3.295	0.798			
CWB 16	3.284	0.754			
CWB 19	3.371	0.677			
CWB 22	3.339	0.772			

Work-related psychological health (WPH 17 items)	Mean	OL^a^	AVE	α	CR
Factor 1: Engagement (4 items)			0.707	0.862	0.906
WPH 04	3.460	0.633			
WPH 09	3.200	0.730			
WPH 20	3.227	0.801			
WPH 21	3.420	0.791			
Factor 2: Stability (4 items)			0.642	0.814	0.877
WPH 01	3.380	0.680			
WPH 02	3.580	0.548			
WPH 11	3.213	0.798			
WPH 12	3.227	0.785			
Factor 3: Fatigue (5 items)			0.902	0.649	0.864
WPH 05	1.847	0.634			
WPH 13	2.033	0.652			
WPH 14	1.773	0.807			
WPH 15	1.760	0.787			
WPH 22	1.933	0.768			
Factor 4: Burnout (4 items)			0.883	0.656	0.824
WPH 16	2.047	0.799			
WPH 17	2.013	0.722			
WPH 18	1.920	0.651			
WPH 19	2.080	0.583			

Mean, the mean score for a specific item; OL, outer loading; AVE, average variance extracted; α, Cronbach’s alpha.

^a^OLs were obtained from confirmatory factor analysis.

**Table 5:** Assessment of discriminant validity for CWB and WPH scales in Survey 2.

Factors^a^	Mean	SD	SWB	PWB	SpWB	Engagement	Stability	Fatigue	Burnout
Clergy well-being (CWB)									
Subjective well-being (SWB)	3.398	0.586	***0.836***						
Psychological well-being (PWB)	3.152	0.645	0.827**	***0.836***					
Spiritual well-being (SpWB)	3.359	0.532	0.738**	0.751**	***0.791***				
Work-related psychological health (WPH)									
Engagement	3.449	0.574	0.794**	0.734**	0.705**	***0.841***			
Stability	3.410	0.527	0.647**	0.628**	0.603**	0.682**	***0.801***		
Fatigue	1.794	0.715	−0.630**	−0.607**	−0.548**	−0.598**	−0.560**	***0.806***	
Burnout	1.906	0.731	−0.539**	−0.514**	−0.451**	−0.476**	−0443**	0.678**	***0.809***

*p < 0.05, **p < 0.01.

Diagonal cells (marked in italic bold) were the square root of the average variance extracted for each factor, while correlation coefficients were reported below.

#### 4.2.3 Measurement invariance

Results showed no significant differences between male and female groups on any CWB or WPH factors (all *p*s > 0.1; see Table 6), confirming the scales’ robustness across gender.

**Table 6:** Known-group validity assessment of CWB and WPH scales in Survey 2.

Variables	Male (*n* = 197) Mean (SD)	Female (*n* = 240) Mean (SD)	*p*-value
Clergy well-being (CWB)			
Subjective well-being (SWB)	3.411 (0.611)	3.388 (0.566)	0.675
Psychological well-being (PWB)	3.169 (0.679)	3.139 (0.617)	0.636
Spiritual well-being (SpWB)	3.345 (0.583)	3.370 (0.487)	0.624
Work-related psychological health			
Engagement	3.475 (0.620)	3.427 (0.534)	0.390
Stability	3.415 (0.575)	3.406 (0.484)	0.863
Fatigue	1.766 (0.704)	1.817 (0.725)	0.466
Burnout	1.857 (0.753)	1.947 (0.712)	0.199

*p < 0.05, **p < 0.01.

#### 4.2.4 Criterion-related and incremental validities explained by WPH and SWB measures

In the baseline model, SWB significantly predicted all four WPH outcomes: engagement (Δ*R*^2^ = 0.630, *p* < 0.01), stability (Δ*R*^2^ = 0.419, *p* < 0.01), fatigue (Δ*R*^2^ = 0.397, *p* < 0.01), and burnout (Δ*R*^2^ = 0.290, *p* < 0.01); fatigue (Δ*R*^2^ = 0.023, *p* < 0.01); engagement (Δ*R*^2^ = 0.018, *p* < 0.01); stability (Δ*R*^2^ = 0.018, *p* < 0.01).

Adding PWB to the model demonstrated significant incremental validity: engagement (Δ*R*^2^ = 0.019, *p* < 0.01), stability (Δ*R*^2^ = 0.027, *p* < 0.01), fatigue (Δ*R*^2^ = 0.023, *p* < 0 01), and burnout (Δ*R*^2^ = 0.015, *p* < 0.01). Finally, inclusion of SpWB further improved model fit: engagement (Δ*R*^2^ = 0 0.018, *p* < 0 0.01), stability (Δ*R*^2^ = 0 0.018, *p* < 0.01), fatigue (Δ*R*^2^ = 0.005, *p* < 0.01), burnout (Δ*R*^2^ = 0.001, *p* < 0.01). These findings confirm H3, demonstrating that the CWB subscales, SWB, PWB, and SpWB, each contribute unique variance in predicting the four-factor WPH model (see Table 7).

**Table 7:** Incremental variance explained by WPH and CWB scales.

Outcomes (WPH scales)	Predicator (CWB)	*β*	*R^2^*	Δ*R^2^*
Engagement	Step1		0.630	0.630**
	SWB	0.794**		
	Step2		0.649	0.019**
	SWB	0.591**		
	PWB	0.246**		
	Step3		0.667	0.018**
	SWB	0.511**		
	PWB	0.150**		
	SpWB	0.214**		
Stability	Step1		0.419	0.419**
	SWB	0.647**		
	Step2		0.446	0.027**
	SWB	0.406**		
	PWB	0.292**		
	Step3		0.464	0.018**
	SWB	0.327**		
	PWB	0.197**		
	SpWB	0.214**		
Fatigue	Step1		0.397	0.397**
	SWB	−0.630**		
	Step2		0.420	0.023**
	SWB	−0.405**		
	PWB	−0.272**		
	Step3		0.425	0.005**
	SWB	−0.363*		
	PWB	−0.222**		
	SpWB	−0.113*		
Burnout	Step1		0.290	0.290**
	SWB	−0.539**		
	Step2		0.305	0.015**
	SWB	−0.360**		
	PWB	−0.216**		
	Step3		0.306	0.001**
	SWB	−0.338**		
	PWB	−0.190**		
	SpWB	−0.059		

*p < 0.05, **p < 0.01.

#### 4.2.5 Common variance bias

Comparisons with the base model revealed no significant differences (CWB: Δχ^2^ = 63.76, *p* > 0.1; WPH: Δχ^2^ = 145.51, *p* > 0.1), suggesting that common method variance did not affect the results. This indicates that the observed relationships in the data are attributable to actual phenomena, not measurement bias. As shown in Table 8, the bifactor model effectively addresses CMV, ensuring the validity of the data’s relationships.

**Table 8:** Assessment of common method variance for CWB and WPH scales.

CWB models	RMSEA	CFI	SRMR	AIC	BIC	Comparison with base model	
						Δχ^2^ (Δdf)	*p*-value
Base	0.045	0.981	0.028	10994.348	11251.384	-	
Single	0.108	0.881	0.056	11493.469	11701.545	523.12 (12)	<0.001
Bifactor	0.062	0.967	0.030	11074.103	11363.778	63.76 (8)	1.000

WPH models	RMSEA	CFI	SRMR	AIC	BIC	Comparison with base model	
						Δχ^2^ (Δdf)	*p*-value
Base	0.042	0.982	0.035	13359.544	13649.22	-	
Single	0.151	0.717	0.096	14449.358	14657.435	1129.81 (20)	<0.001
Bifactor	0.073	0.947	0.040	13511.054	13812.969	145.51 (3)	1.000

χ^2^, chi-square value; df, degrees of freedom; CFI, comparative fit index; SRMR, standardized root mean squared residual; AIC, Akaike Information Criterion; BIC, Bayesian Information Criterion.

#### 4.2.6 The effects of CMB on WPH

**Figure 2:**
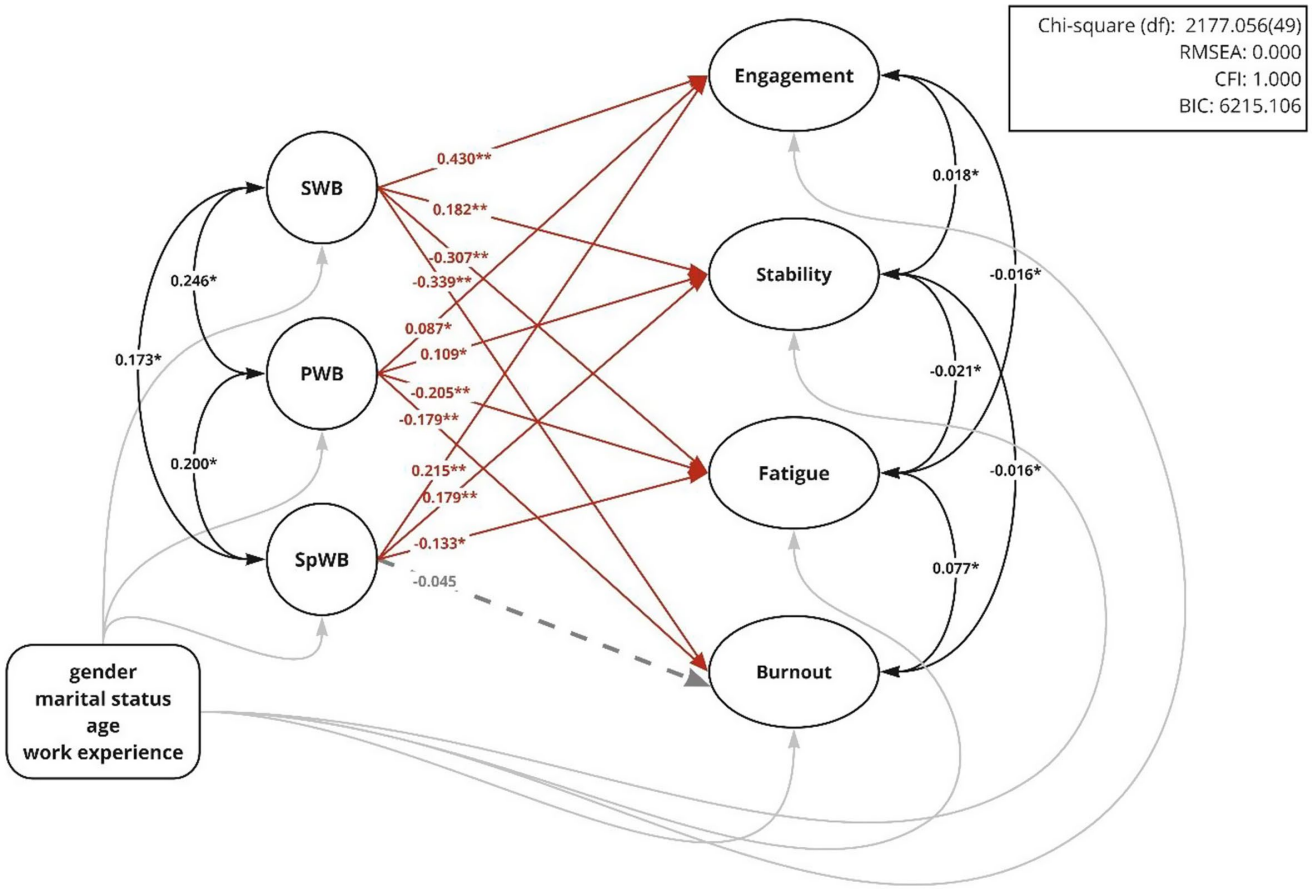
Structural equation model of the effect of CWB dimensions on WPB outcomes. *p < 0 0.05, **p < 0.01.

The SEM results, presented in Figure 2, revealed significant relationships between the CWB and WPH dimensions. The results indicated that SWB significantly enhanced engagement (*β* = 0.41, *p* < 0.01) and stability (*β* = 0.182, *p* < 0.01), while reducing fatigue (*β* = −0.307, *p* < 0.01) and burnout (*β* = −0.339, *p* < 0.01). Furthermore, PWB significantly and positively influenced engagement (*β* = 0.087, *p* < 0.05) and stability (*β* = 0.109, *p* < 0.05), and negatively influenced fatigue (*β* = −0.205, *p* < 0.01) and burnout (*β* = −0.179, *p* < 0.01). Similarly, SpWB significantly enhanced engagement (*β* = 0.215, *p* < 0.01) and stability (*β* = 0.179, *p* < 0.01), while reducing fatigue (*β* = −0.133, *p* < 0.05), but it did not significantly reduce burnout (*β* = −0.045, *p* > 0.1). Higher levels of SWB, PWB, and SpWB improved work-related psychological health among Chinese clergy.

Additionally, the findings reveal that SWB exerts the strongest effect on engagement, followed by SpWB and PWB. SWB and SpWB are equally influential for stability, while PWB has the least impact. All three dimensions of CWB significantly and negatively affect fatigue. SWB remains the most influential factor for burnout, followed by PWB, while SpWB shows no significant impact. Overall, SWB emerges as the most influential factor for all WPH outcomes. SpWB has a more pronounced effect on positive outcomes compared to PWB, whereas PWB demonstrates a stronger influence on negative outcomes.

## 5 Discussion

### 5.1 Discussions of the findings

This study advances understanding of clergy well-being (CWB) by integrating spiritual well-being (SpWB) into established psychological frameworks that traditionally emphasize subjective well-being (SWB) and psychological well-being (PWB) (Diener, 1984; Ryff and Sarason, 1989). Consistent with prior clergy research (Proeschold-Bell et al., 2014; Li, 2005), the findings support a holistic and strength-based conceptualization of well-being that is particularly relevant to faith-based vocations. Empirically, the results confirmed a three-factor structure of CWB and a four-factor dual-spectrum model of work-related psychological health (WPH), encompassing engagement, stability, fatigue, and burnout. Together, these findings validate a multidimensional and occupationally grounded framework for assessing clergy well-being. Moreover, the findings also related to spiritual well-being warrant particular attention.

Cultural differences were interpreted using Hofstede’s national culture framework, which conceptualizes systematic variation in values across dimensions such as individualism–collectivism, power distance, long-term orientation, and indulgence versus restraint (Hofstede, 2001; Hofstede et al., 2010). From an occupational health psychology perspective, this framework is particularly relevant to collectivist cultural contexts such as Chinese society, where occupational well-being is closely tied to role fulfillment, relational harmony, and perceived stability within communal and vocational structures rather than to individual autonomy or self-expression (Markus and Kitayama, 1991; Lu, 2008). Reflecting this orientation, the present model conceptualizes engagement and stability as positive occupational states and treats fatigue and burnout as qualitatively distinct forms of strain rather than as opposite ends of a single continuum. In addition, spiritual well-being (SpWB) is incorporated as a core occupational resource, capturing the embedded role of spirituality in clergy vocational identity, moral responsibility, and resilience (Pargament, 2001; Proeschold-Bell et al., 2014). Finally, the use of experiential and role-based item wording aligns with culturally normative patterns of self-appraisal in Chinese settings and may help mitigate stigma-related response bias in the reporting of psychological distress (Ng, 1997; Hui et al., 2014).

SWB emerged as the strongest overall predictor, underscoring the importance of life satisfaction, emotional balance, and positive affect in sustaining occupational health. This supports the broaden-and-build theory of resilience (Fredrickson, 2001) and aligns with Ryff and Keyes (1995) and Francis et al. (2011). PWB significantly reduced fatigue and increased stability, reflecting the value of purpose, autonomy, and environmental mastery (Ryff and Singer, 1996), though its influence on engagement and burnout was more modest—possibly due to the collectivist context. SpWB promoted engagement and stability by providing existential grounding; however, its non-significant association with burnout may reflect cultural and vocational expectations of spiritual strength and infallibility among clergy. Nevertheless, SpWB remains central to occupational mental health by supporting meaning, transcendence, and connectedness, which are critical resources for sustaining clergy well-being in demanding ministry contexts (Proeschold-Bell et al., 2014; Wong et al., 2017).

Regarding the effect of CWB on WPH, findings showed that SWB was the strongest predictor across all WPH dimensions. This highlights the power of life satisfaction, emotional balance, and positive affect in sustaining occupational health. Clergy, like anyone else, benefit from positive psychological resources. From an occupational health perspective, SWB fosters a broaden-and-build effect (Fredrickson, 2001), expanding cognitive and emotional resources essential for resilience. This finding aligns with Ryff and Keyes (1995) and extends to Francis et al. (2011), who linked SWB with engagement and perseverance in high-demand roles.

PWB also contributed significantly, particularly in reducing fatigue and promoting stability. This supports Ryff and Singer’s (1996) emphasis on self-determination, environmental mastery, and purpose in life. While PWB’s effects on engagement and burnout were less pronounced than SWB’s, this may reflect the influence of relational interdependence in collectivist cultures. Still, PWB provides a strong foundation for sustained growth and adaptability.

### 5.2 Implications

These findings have important implications for clergy care and well-being interventions. The validated 34-item CWB and WPH scale offers a comprehensive tool for assessing both protective factors and potential risks. It provides a culturally sensitive framework that captures the multifaceted nature of clergy well-being.

Interventions to enhance SWB should prioritize improving life satisfaction and emotional balance. Strategies might include promoting work-life harmony, strengthening interpersonal relationships, and addressing culturally specific stressors—particularly relevant in collectivist contexts. To strengthen PWB, training programs can focus on cultivating autonomy, purpose, and resilience. These internal resources help clergy navigate vocational stress more effectively. From an occupational health psychology perspective, such efforts aim not only to reduce symptoms but also to build personal strengths and foster flourishing. Improving SpWB involves addressing cultural barriers such as stigma around seeking help and the expectation of spiritual perfection. Integrating spiritual practices with resilience-building interventions can empower clergy to find meaning in adversity.

To support ongoing self-monitoring, we have launched an online self-assessment tool—A Tool for Self-Screening Chinese CWB and WPH.^2^ This tool offers clergy a private, culturally attuned way to evaluate their current well-being. Research in other cultural contexts also supports the value of age-appropriate, context-sensitive self-assessment tools. For instance, Chen (2021) found that a well-being scale developed for Taiwanese elementary students provided strong psychometric validity and revealed significant developmental differences across grade levels, highlighting the importance of tailored measures. By drawing on the database established in this study, users can obtain personalized scores for both CWB and WPH, helping them identify their position on the occupational psychological health spectrum. Religious organizations and seminaries can incorporate this tool into broader support systems, including regular well-being assessments, counseling services, peer support networks, and leadership development focused on stress management and emotional resilience. These initiatives reflect the orientation of occupational health psychology and resonate with core cultural values such as relational harmony and communal care (Lu, 2008).

### 5.3 Limitations and future research

Despite its contributions, this study has several limitations that should be acknowledged. First, the sample consisted of Chinese-speaking clergy rather than the general population. Given the practical constraints of recruitment and the low feasibility of accessing clergy in mainland China, data were collected exclusively from clergy serving in Taiwan. This geographic concentration limits the generalizability of the findings to other Chinese-speaking contexts.

Second, the findings should be interpreted in light of the sample’s religious and denominational characteristics. Participants were exclusively Christian clergy recruited through national Christian church networks in Taiwan, with the sample predominantly comprising Protestant clergy across multiple denominations. Catholic clergies were not included. This denominational scope is relevant because differences in vocational structure, marital norms, and institutional support across religious traditions may shape experiences of well-being and work-related psychological health in distinct ways. Accordingly, caution is warranted when generalizing the findings beyond Protestant Christian clergy. Future research should explicitly examine denominational and interfaith differences to further refine clergy well-being assessment.

Beyond sampling considerations, several additional limitations merit attention. First, the cross-sectional design precludes causal inferences. Longitudinal or intervention-based studies are needed to examine changes in clergy well-being and work-related psychological health over time. Second, reliance on self-report measures may introduce social desirability bias, particularly in religious contexts where the expression of distress may be stigmatized. Future studies could incorporate peer assessments, supervisor evaluations, or qualitative approaches to obtain a more comprehensive understanding.

Third, although spiritual well-being was incorporated as a core construct, the SpWB measure may not fully capture the diversity of theological perspectives within Chinese-speaking Christian contexts. Future research should explore how theological traditions and denominational practices shape spiritual well-being and its relationship with occupational health. Finally, while the dual-spectrum model of work-related psychological health demonstrated strong empirical support in this study, its generalizability remains limited. Further validation across other Chinese-speaking regions, such as Hong Kong and Southeast Asia, as well as among other helping professions, would strengthen its applicability.

Future research may also examine whether targeted interventions based on subjective, psychological, and spiritual well-being can improve clergy’s occupational health in real-world settings. Additionally, exploring individual differences, such as personality traits, may help refine well-being interventions. Such efforts align with a person-centered and strengths-based approach to mental health, enhancing both personal and vocational flourishing.

## 6 Conclusion

This study makes significant theoretical and practical contributions to the understanding of clergy well-being, particularly within the unique cultural context of Chinese society. By integrating SWB, PWB, and SpWB into a unified framework, it advances a culturally grounded model for assessing and promoting clergy’s occupational health. The findings highlight how these dimensions distinctly and interactively influence key work-related psychological health, including engagement, stability, fatigue, and burnout. This offers a nuanced perspective that moves beyond traditional Western paradigms.

Of particular importance is the central role of SWB, which emerged as the most influential factor across well-being outcomes. This underscores its foundational relevance in collectivist cultures, where emotional harmony and life satisfaction are highly valued. The complementary influence of PWB further reinforces the importance of inner psychological strengths such as purpose, autonomy, and mastery in building vocational resilience. By bridging the principles of occupational health psychology with cultural sensitivity, this study provides a foundation for developing holistic, strengths-based interventions tailored to the lived experiences of clergy.

## Data Availability

The raw data supporting the conclusions of this article will be made available by the authors, without undue reservation.

## Footnotes

1
oursweb.net

2
http://www.jaimeicare.com.tw
