# Correction: Real-world evidence for clergy well-being: developing a culturally grounded self-screening tool to promote sustainable mental health outcomes

**DOI:** 10.3389/fpsyg.2026.1823085

**Published:** 2026-04-08

**Authors:** Wen-Cheng Li, Yu-Ting Chin, Li-Hsin Wu, I-Ling Ling, Chien-Hung Lee

**Affiliations:** 1 Department of Public Health, College of Health Sciences, Kaohsiung Medical University, Kaohsiung, Taiwan; 2 Department of Psychology, University of Toronto, Toronto, ON, Canada; 3 Department of Marketing & Tourism Management, National Chiayi University, Chiayi, Taiwan; 4 Research Center for Precision Environmental Medicine, Kaohsiung Medical University, Kaohsiung, Taiwan; 5 Department of Medical Research, Kaohsiung Medical University Hospital, Kaohsiung Medical University, Kaohsiung, Taiwan

**Keywords:** Chinese clergy, clergy well-being, psychological well-being, scale development, spiritual well-being, subjective well-being, work-related psychological health

Affiliation “Department of Public Health, College of Health Sciences, Kaohsiung Medical University, Kaohsiung, Taiwan” was omitted for author Yu-Ting Chin. This affiliation has now been added for author Yu-Ting Chin.

Author Yu-Ting Chin was erroneously assigned to affiliation “Department of Marketing & Tourism Management, National Chiayi University, Chiayi, Taiwan.” This affiliation has now been removed for author Yu-Ting Chin.

A correction has been made to Section “4.2.4 Criterion-related and incremental validities”:

“fatigue (Δ*R*^2^ = 0.397, *p* < 0.01), and burnout (Δ*R*^2^ = 0.290, *p* < 0.01); fatigue (Δ*R*^2^ = 0.023, *p* < 0.01); engagement (Δ*R*^2^ = 0.018, *p* < 0.01); stability (Δ*R*^2^ = 0.018, *p* < 0.01).”

The original version of this article has been updated.

