# Supplementary material for: Real-world evidence for clergy well-being: developing a culturally grounded self-screening tool to promote sustainable mental health outcomes
Source: Front Psychol. 2026 Mar 3;17:1677071. doi: 10.3389/fpsyg.2026.1677071 (PMC12991992; doi:10.3389/fpsyg.2026.1677071)
Supplement: Supplementary file 1 [file Supplementary_file_1.pdf]

Supplementary Materials

Related article:
Real-World Evidence for Clergy Well-Being: Developing a Culturally Grounded Self-Screening Tool to Promote Sustainable Mental Health Outcomes

Wen-Cheng Li, Yu-Ting Chin, Li-Hsin Wu, I-Ling Ling*, Chien-Hung Lee*

Frontiers in Psychology.

DOI: 10.3389/fpsyg.2026.1677071

The following content provides full items and scoring instructions for the Scale of Chinese Clergy Well-Being (CWB) and Work-related Psychological Health (WPH). All items are rated on a 4-point *Likert* scale (1 = strongly disagree, 2 = disagree, 3 = agree, 4 = strongly agree).

Supplementary Appendix A: Scale of Chinese Clergy Well-Being (CWB).
Supplementary Appendix B: Scale of Chinese Clergy Work-related Psychological Health (WPH).

Scale of Chinese Clergy Well-being

Well-being Questions	strongly disagree	disagree	agree	strongly agree
1. I often feel the presence and power of God.	①	②	③	④
2. I often feel God's will and guidance in my life.	①	②	③	④
3. I enjoy my life.	①	②	③	④
4. I feel that life is meaningful and purposeful.	①	②	③	④
5. My job brings me a sense of accomplishment.	①	②	③	④
6. I often feel an excellent relationship with God.	①	②	③	④
7. When leading the worship, I feel the power of God's presence.	①	②	③	④
8. In different gatherings, I often experience the presence of God.	①	②	③	④
9. I feel full of vitality.	①	②	③	④
10. I am optimistic about the future.	①	②	③	④
11. When dealing with crisis events among friends, I often sense the presence and assistance of God.	①	②	③	④
12. I understand the meaning of life.	①	②	③	④
13. I perceive the love and presence of God when administering the sacraments.	①	②	③	④
14. I like myself.	①	②	③	④
15. I am an attractive person.	①	②	③	④
16. In my life, I often experience the presence and power of God.	①	②	③	④
17. I feel safe.	①	②	③	④

Scoring	Corresponding Questions	Total*
**Subjective Well-being**	3, 4, 5, 12	
**Psychological Well-being**	9, 10, 14, 15, 17	
**Spiritual Well-being**	1, 2, 6, 7, 8, 11, 13, 16	

*Calculate the score by summing the corresponding item scores. Higher scores indicate higher levels of the respective construct.

Scale of Chinese Clergy Work-related Psychological Health

Work-related Psychological Health Questions	strongly disagree	disagree	agree	strongly agree
1. I have established good relationships with fellow members.	①	②	③	④
2. I feel honored by my service work.	①	②	③	④
3. I take pleasure in my service work.	①	②	③	④
4. Putting in my best effort to serve, yet not experiencing much sense of achievement, I find myself reducing my level of commitment.	①	②	③	④
5. I am optimistic about my ministry.	①	②	③	④
6. Fellow members appreciate the quality of my service.	①	②	③	④
7. I am respected by fellow members.	①	②	③	④
8. Facing the current service-related issues makes me feel lost.	①	②	③	④
9. Due to the lack of variety in the service tasks, the job has become mundane and unexciting.	①	②	③	④
10. I am unable to derive a sense of accomplishment from my service work.	①	②	③	④
11. My workload is heavy and overwhelming.	①	②	③	④
12. There are many conflicts among organizational members and my coworkers.	①	②	③	④
13. Faced with pressure, I sometimes find it difficult to breathe.	①	②	③	④
14. I feel powerless and unable to live up to my expectations.	①	②	③	④
15. I am passionate about the work of the preacher.	①	②	③	④
16. I am honored in my ministry job.	①	②	③	④
17. I am not satisfied with the current working situation.	①	②	③	④

Scoring	Corresponding Questions	Total*
**Engagement**	3, 5, 15, 16	
**Stability**	1, 2, 6, 7	
**Fatigue**	4, 8, 9, 10, 17	
**Burnout**	11, 12, 13, 14	

*Calculate the score by summing the corresponding item scores. Higher scores indicate higher levels of the respective construct.
